# Barriers and recruitment strategies for precarious status migrants in Montreal, Canada

**DOI:** 10.1186/s12874-019-0683-2

**Published:** 2019-02-26

**Authors:** Margaux Fête, Josephine Aho, Magalie Benoit, Patrick Cloos, Valéry Ridde

**Affiliations:** 1 University of Montreal Public Health Research Institute (IRSPUM), Montreal, Canada; 2 School of Public Health, University of Montreal, Montreal, Canada; 3 School of Social Work, Faculty of Arts and Sciences, University of Montreal, Montreal, Canada; 4 French Institute for Research on Sustainable Development (IRD), CEPED (IRD-Université Paris Descartes), Universités Paris Sorbonne Cités, ERL INSERM SAGESUD, Paris, France; 5 Fellow de l’Institut Français des Migrations, Paris, France

**Keywords:** Precarious status migrants, Recruitment strategies, Research method, Research participation, Hard to reach population

## Abstract

**Background:**

Precarious status migrants are a group of persons who are vulnerable, heterogeneous, and often suspicious of research teams. They are underrepresented in population-based research projects, and strategies to recruit them are described exclusively in terms of a single cultural group. We analyzed the recruitment strategies implemented during a research project aimed at understanding precarious status migrants’ health status and healthcare access in Montreal, Canada. The research sample consisted of 854 persons recruited from a variety of ethnocultural communities between June 2016 and September 2017. This article analyzes the strategies implemented by the research team to respond to the challenges of that recruitment, and assess the effectiveness of those strategies. Based on the results, we share the lessons learned with a view to increasing precarious status migrants’ representation in research.

**Method:**

A mixed sequential design was used to combine qualitative data gathered from members of the research team at a reflexive workshop (*n* = 16) and in individual interviews (*n* = 15) with qualitative and quantitative data collected using the conceptual mapping method (*n* = 10).

**Results:**

The research team encountered challenges in implementing the strategies, related to the identification of the target population, the establishment of community partnerships, and suspicion on the part of the individuals approached. The combination of a venue-based sampling method, a communications strategy, and the snowball sampling method was key to the recruitment. Linking people with resources that could help them was useful in obtaining their effective and non-instrumental participation in the study. Creating a diverse and multicultural team helped build trust with participants. However, the strategy of matching the ethnocultural identity of the interviewer with that of the respondent was not systematically effective.

**Conclusion:**

The interviewers’ experience and their understanding of the issue are important factors to take into consideration in future research. More over, the development of a community resource guide tailored to the needs of participants should be major components of any research project targeting migrants. Finally, strategies should be implemented as the result of a continuous reflexive process among all members of the research team.

**Electronic supplementary material:**

The online version of this article (10.1186/s12874-019-0683-2) contains supplementary material, which is available to authorized users.

## Background

### Problem

More than ever, migration has become a global phenomenon, and the number of vulnerable migrants is growing. Precarious status migrants are a heterogeneous category of persons who have no legal permanent status (asylum-seekers, temporary workers, foreign students, live-in caregivers, victims of human trafficking, persons from moratorium countries or undocumented) [1]. The precariousness of migratory status is a potentially deleterious factor, to the extent that it may entail difficult living and working conditions [1]. Some authors have also noted that the rapidity of deterioration in migrants’ health status over time may depend on their country of birth [2].

However, there is a lack of information regarding healthcare access and health status, as well as other determinants of health, for precarious status migrants [3]. In fact, since the majority of published studies have been conducted in healthcare centres, there are few population-based studies to provide evidence that is representative of the migrant population in its diversity [3]; yet such studies are needed to reduce the inequalities in health and access to care that these migrants face [1]. Moreover, precarious status migrants are a population that is vulnerable, heterogeneous, and difficult to reach for purposes of research and intervention [3]. As a result, researchers face many barriers related to identifying, accessing, recruiting, and collecting data from these minority groups [4, 5]; consequently, they are underrepresented in studies [5–8].

In addition, researchers encounter language and cultural barriers [9–12], as well as difficulties in contacting and following up with persons who are often mobile [13, 14]*.* Authors have also identified lack of interest and fears regarding the consequences and benefits of research as a challenge [15, 16]. Suspicion towards research teams is all the more present among migrants without status, who fear that participating in studies could lead to disclosure of their migratory status, being reported to the authorities, or experiencing discrimination [17, 18]*.*

Given that it is not practicable to use traditional sampling methods based on a list of persons, specific methods need to be put in place to reach them [19–21]. Some researchers use venue-based sampling [22], which involves conducting a preliminary study of the geographic areas in which there is a high proportion of the target population [23]. Other researchers use the respondent-driven sampling method [24, 25], in which respondents are recruited directly by their peers [26, 27], or the snowball method, in which participants are identified by others who have already participated in the study [28]. Partnerships between community members and research teams may also be helpful in accessing the target population [11, 14, 29, 30]*.* In this setting, the most frequently used recruitment strategies involve setting up a multicultural team and translating information and recruitment materials into the languages of the target communities [14, 31–34].

To our knowledge, in the literature there are only three reviews [8, 19, 35] and two interventional studies [13, 36] that have identified recruitment strategies for use in a diverse setting of migrants. Developing research recruitment strategies, however, requires a good understanding of this population’s diversity, at both the cultural and individual levels, in terms of people’s migration pathways, social status, and level of acculturation [37, 38].

### Context of the present study

Montreal is a city of 1.9 million inhabitants in the province of Quebec (Canada), in which are represented 180 nationalities and a variety of religions, migratory pathways, and levels of social integration [39]. There are numerous community centres, some of which attempt to represent these countries or regions of the world. These large groups, called ‘communities’, refer to populations or organizations offering services, and are both part of, and the product of, social construction processes. The notions of ‘race’, ‘ethnicity’, ‘culture’, ‘nationality’, or even ‘community’ are often confounded, both in the urban and political spheres and in the scientific field. Yet the discourse on *difference* (for example, cultural) is often much too static and tends to ignore the fluidity of identities and practices, as well as its social grounding. Moreover, this type of discourse is entrenched in power relationships that can have harmful consequences on individuals and groups [40–42]. It is therefore precisely by seeking to understand power relationships and their social consequences (discrimination and differential access to resources and opportunities, including healthcare) that we have attempted to draw a diversified portrait of Montreal’s migrant population on the basis of national, geographic, and cultural origins. We therefore decided to ensure a certain level of national and geographic representation of the precarious status migrant population by identifying and contacting the community centres in the Montreal region. We put in place recruitment strategies for a dozen communities (African sub-Saharan, Anglo-Caribbean, Chinese, European, American (United States), Haitian, Latin-American, Maghrebin, Middle-Eastern, Russian, South Asian, and West Balkan), covering the majority of ethnicities most represented in Montreal. In 2016, before the study was launched, several barriers and recruitment strategies were highlighted during the research planning phase based on interviews and discussion groups with key informants from the different communities [43]. A multicultural and multidisciplinary team of interviewers was created and trained. With a view to producing data on precarious status migrants’ health status, healthcare access, and determinants of health, two types of recruitment were conducted between June 2016 and September 2017: in a population-based recruitment (436 persons recruited) and in a healthcare setting-based recruitment, the *Médecins du Monde* (Doctors of the World) clinic (370 persons recruited) [44]. For the population-based recruitment, the research team chose mainly to use a venue-based sampling methodology, coupled with snowball sampling in certain communities, as well as with an active campaign in social media, in the different communities, in the local press (print and radio), etc. The interviewers, as community experts, were invited to make suggestions, but the project coordinator was responsible for the final decision regarding recruitment venues. While the respondent-driven sampling method has also been used to reach migrants in some contexts [24], it requires that the targeted persons be networked among themselves, which was not the case for our target population in Montreal. Therefore that method was not selected. Thus, the team put in place collaborations with community organizations and places of worship and recruited primarily in public places. The required sample size for the population-based part of the recruitment was set at 400 participants to be able to estimate the prevalence of unmet health needs. The fact that the initial objective for the study, to recruit 400 persons in the urban area, was achieved suggests that the strategies were effective. It is important to analyze reflexively [45] the strategies implemented for the population-based (or community-based) recruitment.

### Objectives of the study

This study had two objectives: 1) to analyze the strategies implemented by the research team to respond to the challenges of that recruitment, and assess the effectiveness of those strategies; and 2) to share the lessons learned by the interviewers (interviewers, coordinators, trainees) who recruited migrants, with a view to increasing the representation of precarious status migrants in quantitative healthcare studies.

## Methodology

### Analysis frameworks

We used two frameworks to collect and analyze the data. Based on a systematic literature review, Bonevski et al. [4] summarized the main barriers to sampling, recruitment, and participation of hard-to-reach populations: suspicion about the study and the research team; fear of authorities; perception of negative impacts; fear of mistreatment and exploitation; fear of stigmatization; absence of benefits; lack of awareness about the research; issues of gender and age; and poor response rates. Even if strategies are implemented to overcome these barriers, those strategies, too, take place in different contexts [46]. For this reason, we decided also to use Pawson’s conceptual framework on the dimensions of context [47]. In fact, the strategies relate to the interviewers (individual context), but also to interpersonal relationships among the team members and with the precarious status migrants (interpersonal context). Lastly, they relate to the research setting (institutional context) and to the social and cultural environment of the organization (infrastructural context).

### Design and data collection tools

**Fig. 1:**
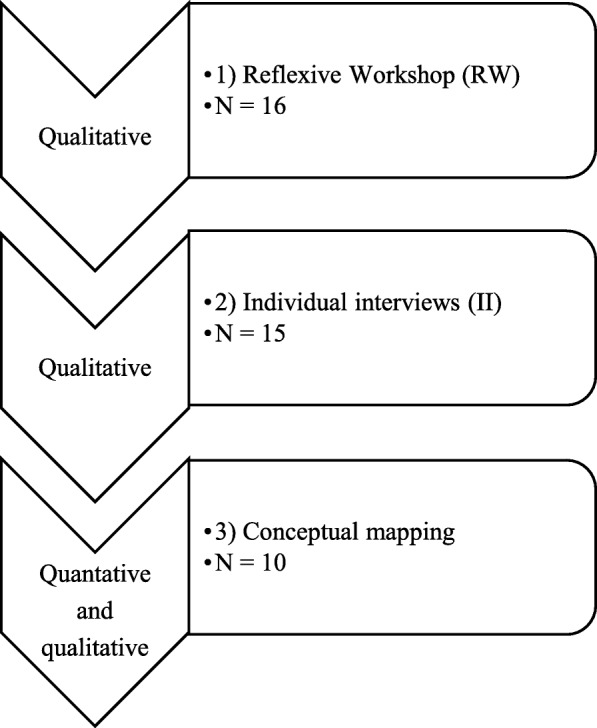
Sequential mixed analysis design

We used a mixed sequential research design with three phases, in which the results of each phase contributed to the development of the next phase [48] (Fig. 1). The results of phases 1 and 2 were used to analyze recruitment strategies, whereas the results of phase 3 highlighted lessons learned by the interviewers.

The reflexive workshop (RW) was held on August 3, 2017, for 3 h. The purpose of a reflexive workshop is to enable participants to produce a retrospective and reflexive analysis of an action implemented. In the present case, this involved allowing participants to reflect on the recruitment process, analyse the processes, and draw lessons from them. The reflexive approach is an important process in public health [49]. During this workshop, open discussion reflections were focused on community-based recruitment strategies and lessons learned, as well as on the classification of recruitment venues based on their relevance. The interviewers still in service (*n* = 22) were invited, and 16 participated (72%). Notes were taken and the discussions were fully transcribed and analyzed. Individual interviews (II) were conducted between September 18 and October 29, 2017, lasting 1.5 to 2 h. The semi-structured interview grid was adjusted following pilot interviews conducted with two interviewers. The content of the interview corresponded to the dimensions of the two analysis frameworks of Bonevski [4] and Pawson [47]. Notes were taken and statements were fully transcribed and analyzed. The interviewers who participated in this research were selected based on a contrasted heterogeneous sample representative of the full set of interviewers for maximum diversification of viewpoints. Table 1 presents the characteristics of the population of interviewers (*n* = 41) and of the sample (*n* = 15) who were interviewed. The interviewers who participated in the present study came from diverse backgrounds (social work, intercultural mediation, psychology, public health, law, and politics). Most already had experience working with vulnerable populations, and some interviewers were in contact with persons without status, either in their work or through volunteer involvement with local associations. One interviewer had significant experience as a research assistant working with minors in precarious situations, but for most, this was their first research work experience in Canada. The majority of the interviewers were themselves migrants with very diverse pathways.

**Table 1:** Characteristics of interviewers participating in individual interviews

	N sample	N total
Participants	15	41
Men	7	25
Women	8	16
Region of origin		
South America	1	2
Central America	1	8
North America	2	2
Caribbean	2	3
West Balkans	1	2
Western Europe	2	6
Central Asia	1	3
South Asia	2	4
Maghreb	2	5
Sub-Saharan Africa	1	6
Length of involvement in the project		
1 month	0	2
4–5 months	5	27
8 months	2	2
14 months	7	9
24 months	1	1

The third and final step consisted of a conceptual mapping workshop based on a methodology presented elsewhere [50]. This four-hour workshop involved a quantitative and qualitative group consensus method. It was held on November 23, 2017. The interviewers present in Montreal at the time of the workshop (*n* = 20) were invited to participate. There were 10 participants (50%), and all had previously taken part in individual interviews and the reflexive workshop. The reason why not all of the 16 reflexive workshop participants attended the concept mapping workshop was that, by the time of the concept mapping workshop, the interviewers were either no longer employed in the study, or had work or family obligations that prevented them from attending.

At the concept mapping workshop, in response to the question, “*To ensure the success of a survey among precarious status migrants, it would be important to*…”, participants produced a list of 94 statements. Those statements were then printed and distributed to the participants as a list. They were asked to give each statement two scores, on a scale of 1 to 5 (5 being the most important), for relevance and feasibility. Then each participant organized the 94 statements into categories that made sense to them. Six of the participants were women, and participants’ different geographic regions of origin, as well as the different statuses within the project (trainee, interviewers, coordinator), were represented.

### Data analysis

The analysis process followed a convergent synthesis design with integration of results. To integrate the qualitative and quantitative results, we combined two methods. On one hand, we used the data comparison method [51] by highlighting similarities or differences. On the other, we used the data assimilation method by merging the qualitative and quantitative data into themes [52]. For the qualitative data analysis performed using QDA Miner, we used the framework analysis method [53] combined with the inductive and deductive approach, which consists of developing the analytical framework for coding based on both the conceptual framework and emerging themes [54]. QDA Miner is a software program for managing and coding qualitative data to support analysis. The data from the concept mapping were entered into the *Provalis Research* software program. Quantitative analyses (multidimensional scaling, hierarchical cluster analysis [50]) were used to produce a collective map with all the participants’ statements. Two statements were reclassified and eight clusters were identified; names were assigned to these by the researchers and then validated via email by 50% (5/10) of the interviewers having participated in the present study. The clusters presented a very homogeneous relevance score, with a standard deviation very close to zero (= 0.1359). As such, we examined the links between the relevance scores and the feasibility scores of the different clusters.

### Ethical considerations

For the interviews, we adapted to the interviewers’ needs by being flexible with regard to interview location and by respecting certain participants’ wish not to have their interviews recorded. They were given a compensation of 20 CAD at the end of each interview. They were free to respond or not, and to withdraw from the study at any time. The interviews were anonymous. This study of interviewers was part of a study of migrants that was approved by the ethics committee of the University of Montreal Hospital Research Centre (CRCHUM) (14.204).

## Results

### Challenges

#### Difficulty in identifying and accessing uninsured migrants

One of the major challenges encountered had to do with identifying the target population, as it is very homogeneous and its members are not distinguishable from the other members of their community: *“The fact that we were looking in all the communities, that was the greatest challenge, because we didn’t know how to identify those persons…. They are a very diverse [group of] people”* [woman, Canada, II]. It was sometimes difficult to identify the venues and times for recruitment when the target population would be present: *“Sometimes we would attend events, we’d stay the whole day, there was no one; these are the uncertainties of research, we can’t know who we’ll meet”* [man, Haiti, II]. Moreover, the statements relating to the identification of key informants in the community presented a high level of disparity between their relevance and feasibility scores in the conceptual map (> 1) [Additional file 1]. In fact, the interviewers noted the time and effort required to identify key informants.

#### Challenges relating to resources and logistics on the ground

According to some interviewers, more human resources would have been needed, particularly for the project’s communications strategy (social media, community media, social marketing) and the linkage with key informants and community organizations: *“I think it would require someone in charge of communications and partnerships full-time”* [RW]. As well, adapting to people’s preferences in terms of interview location required flexibility on the part of the interviewers, as well as budgetary resources, with some people not presenting at all, or arriving late to the scheduled meeting. Lastly, climatic conditions, lack of time and interest among the persons approached, and the transporting and management of materials may sometimes have influenced interviewers’ motivation during recruitment activities. In the concept mapping, the interviewers highlighted the importance of a having a strategy to stay motivated and to plan their time in the field.

#### Suspicion and fear of stigmatization

The interviewers often spoke about the fear of denunciation among the people encountered, and their fear that their status could be exposed, whether in public places, community celebrations, places of worship, or community cafes: *“They would say to me: but can I trust you? Aren’t you working for Immigration?”* [woman, Algeria, II]. In some venues that supported people’s anonymity, or those where people were in a frame of mind to accept help, people seemed more at ease. On the other hand, in some places of worship, people were afraid their status would be exposed to their peers: *“When we approach people in places of worship, they don’t really need help, but when they’re in the sharing store [food bank] or the Médecins du Monde clinic, they’re in a frame of mind where they need help… if I had met them in the street, they surely wouldn’t have told me the truth”* [woman, India, II].

#### Issues related to community partnerships

While the concept mapping clusters did not generally present any great differences between their relevance and feasibility scores (< 0.5), the cluster entitled “Recruitment tailored to settings and communities”, which refers mainly to the linkages with community organizations and places of worship, stood out because of the large discrepancy between its relevance score (4.17) and its feasibility score (3.42) [Additional file 2]. This reflects the challenges interviewers faced in establishing reciprocal collaboration to gain access to the organizations. Some community members saw no benefit in collaborating with the study, as they were unaware of the difficulties in accessing care associated with migrant status. In addition, some community or religious leaders were suspicious and preferred to protect their members from the study: *“They won’t necessarily refer their members to you, because they are protecting them”* [man, Colombia, II].

#### Issues related to culture and gender

For reasons specific to the different cultures and to individuals, interviewers noted a certain suspicion among migrants when approached by someone from their own cultural community. For example, *“In Albania, we trust what strangers tell us, but not an Albanian. So I think it would have been very easy for a French person, a German, or an Italian to recruit these people, but not for an Albanian”* [woman, Albania, II]. Gender issues were identified by a small number of interviewers, and particularly in the Muslim community, such that some interviewers suggested matching the genders of interviewer and interviewee: *“The imam did not accept to meet with me, because he can’t talk with a woman. After several phone calls, he said to me: Yes, you can come here, but only with your husband!”* [woman, Albania, II].

#### Ethical challenges

The individuals encountered sometimes expressed their fear of being research subjects: *“Sometimes people think you want to do a study on them. They imagine laboratory studies”* [man, Senegal, II]. The interviewers also faced challenges relating to the compensation in some communities. In the African (sub-Saharan) community, particularly among the Senegalese, for example, it may have aroused suspicion. In the Middle-Eastern community, particularly among Iranians, it may have offended their pride: *“Putting too much emphasis on the 20 dollars doesn’t help, because they’re too proud”* [RW]. Moreover, the compensation may sometimes have introduced biases in terms of data quality, as in the case where one leader of a community organization referred members with the sole aim of collecting compensation. Lastly, the participants’ complex situations, whether in terms of their health status or their migratory pathway, often caused interviewers to feel powerless: *“The people we meet are sometimes in very precarious situations, because they’re not in contact with resources, because they have nothing, and we feel powerless…”* [woman, Canada, II]. Thus, providing support and guidance to interviewers would seem to be an inherent challenge in research projects on precarious status migrants.

### Strategies

Table 2 presents the various strategies to be implemented in response to the barriers identified. In the following text, we develop the main strategies implemented that might have had the greatest impact, according to the interviewers.

**Table 2:** Most promising strategies and lessons learned in response to barriers identified

Barriers	Interpersonal strategies	Individual strategies	Institutional strategies	Infrastructural strategies
Adapting the research to participants	Administer the questionnaire in the presence of an interviewer who can restate and explain certain questions	Present the project material to business people and religious leaders (in language that is simple and accessible to all) Have the research materials on hand to distribute when attending events	Use project materials that are appealing as well as linguistically and culturally appropriate	
Difficulties in identifying and accessing the target public	Promote the sharing of information (mail/reports at regular intervals)	Have interviewers who are motivated, engaged, and available to work non-standard hours	Involve interviewers and members of the community and the target population in selecting the strategies	Hold regular reflective meetings among all team members Focus on neighbourhoods with a high density of migrants and on highly affluent areas.
Suspicion towards the research team	Foster an approach that is collaborative and reciprocal, with personalized follow-up over the long term with community members	Have a diversified team of interviewers with good interpersonal skills	Implement strategies to identify the team and for communications Set up a phone line to be reachable at all times Make regular visits into the field	Focus on venues where people are in a frame of mind to be receptive to help, or where there is an environment of privacy and anonymity Adapt recruitment schedules to the peak hours of the target population, particularly during community festivals
Ethical issues related to recruitment		Have interviewers who have had experiences with vulnerable populations, and who are empathic and good listeners Provide reassurance and minimize the impact of certain stigmatizing questions (housing, habitat) Verify the consistency of responses during data collection Talk about the long-term benefits of the program	Train interviewers Have a guide available to community resources adapted to people’s need Put questions about migratory status at the end of the questionnaire	Give participants a choice regarding the location and the interviewer
Issues related to culture and gender	Socialize and learn about the other members of the team	Have mixed teams in the field (gender, culture, language, age) and bilingual interviewers		Use a targeted approach for certain communities that are less inclined to respond in the public space
Logistic challenges in the field		Use simple language adapted to the people encountered	Define the role of the field coordinator (as motivator and time manager) Set up visible and attractive kiosks, and fun activities to attract the attention of participants (balls, games)	

#### Interpersonal strategies

- Building relationships of trust with community members

The research team adopted a collaborative approach and established reciprocal relationships with community members (religious leaders, business people, leadership of community organizations). This was achieved by providing training and a guide to resources, as well as by team members’ attendance at community activities. Personalized reporting, including face-to-face meetings and regular contact over the long term, worked better than emails and phone calls. *“I noticed that visiting in person worked much better, was much more effective. Phone calls and emails don’t work”* [woman, Canada, II]. Follow-ups, regular visits, information sharing, and initial contact before recruitment began helped establish links with community members.

- The importance of having the questionnaire administered by an interviewer

Having the questionnaire administered in the presence of an interviewer made it possible to restate the questions and to take into account the different nuances of language within a same community (particularly in the cases of Maghreb and India, for example) and to verify the consistency of responses. In the conceptual mapping, the interviewers classified the statement “Use a diversity of means (telephone, Internet) to respond to the questionnaire” among the 10 least relevant [Additional file 3]. It therefore appeared crucial that an interviewer be present to administer the questionnaire: “*Those people with absolutely no schooling, they didn’t understand [the questionnaire] at all, neither in their own language nor in English. So, on the Internet, I don’t know to what extent they would be able to answer the questions correctly”* [RW].

#### Individual strategies

- A team that is representative of the differences

First, one of the main recruitment strategies was the creation of a multicultural team. This strategy was adopted to contend with the language and cultural barriers, as well as with participants’ suspicion, revealed in the survey preparation [43]. In practice, the sharing of a common culture between interviewers and participants was a facilitating factor that overcame language barriers and allowed people to express themselves confidently in their native language. The sharing of common ethnocultural identity between the interviewer and the person recruited was not always enough to establish a relationship of trust, as some people preferred to be interviewed by someone from outside their community. Second, the sharing of experiences relating to migratory status or pathways, as well, sometimes lifted the barriers of suspicion or fear of judgment and facilitated recruitment: *“I realized that I had lived through the same thing, because when I had no papers, I was afraid of nearly everyone, because I thought everyone would denounce me. So maybe this is what helped me; I knew how to approach someone who was afraid to talk”* [man, Senegal, II]. It was therefore useful for the research team to be familiar with factors associated with migratory status.

- A team with professional and interpersonal skills

It seemed important that the interviewers should already have had experience with vulnerable populations so they would be prepared to contend with the complex situations they would encounter. In addition, the interviewers indicated that it was important to guarantee the anonymity of the people encountered and to reassure them regarding the confidentiality of the data. By not adopting a posture of authority or judgment, they were able to reassure the participants, particularly in relation to questions considered sensitive for the interviewers (habitat, migratory status). Lastly, it seemed important to let people choose the location and date of the interview, or to let them call back on the project’s phone line: *“Leaving the choice to the person, that makes all the difference between a yes and a no, because people feel they have control”* [RW]. The fact that 40% of the 10 most relevant statements in the conceptual mapping were from the cluster entitled “Interviewers who are expert and engaged” reinforces the necessity of hiring nterviewers who have expertise regarding vulnerable populations or migrants with precarious status [Additional file 4]. In this cluster, numerous statements refer to the human qualities of the research team, such as empathy, listening, and the capacity to adapt to people’s characteristics (age, personality, culture).

#### Institutional strategies

- Creating a guide to resources adapted to people’s needs

According to the interviewers, financial compensation was not a key motivational factor, and even if it is considered to be fair compensation, it must be kept in mind that it can have a negative impact on some people. However, talking about the short-term benefits of the research project (such as the guide to community resources) or the positive consequences over the long term had a positive impact on recruitment: *“Telling them that we could offer things to people right away, such as services, resources… they’re more willing to listen, because they know they’ll get something in return”* [RW]. The cluster entitled “Focusing on participants” [4.05; 3.86] refers primarily to the fact that participants were given tools that were practical and adapted to their needs [Additional file 1].

- Project visibility in the community and cultural adaptation of recruitment materials

The use of community media and social networks, as well as of a strategy to identify the research team during field outings (banners, cards, information kiosks, and photos of team members on social networks) gave the team visibility and reinforced a sense of trust in the community: *“There was also the fact that they had seen us everywhere, a kind of saturation; people said, ‘I heard you on the radio, I saw you at such-and-such an event.’ Given that you’re everywhere, what you’re doing must be serious”* [woman, Canada, II]. To reach the communities, it was important to select the appropriate media for each of them. For example, Facebook was especially effective for reaching the Latin-American community [Additional file 5]. Numerous statements in the cluster entitled “Social marketing of the survey” [4.3; 4.05] [Additional file 5] refer to the importance of adapting the communications strategy to the various communities to make the project’s issues and the questionnaire accessible. For purposes of cultural and linguistic adaptation, it was helpful that the interviewers were involved in the translation of the materials. Some interviewers also suggested that closer collaboration between the researchers and the interviewers would have been beneficial in developing the questions for both the questionnaire and the study, as well as the promotional materials about the project.

#### Infrastructural strategies

- Using approaches specifically tailored to the targeted communities and contexts

It was clearly advisable to consider cultural habits and preferences when implementing recruitment strategies and selecting recruitment locations. For example, the snowball technique worked well enough in the Latin-American community, but less well in others where people were more suspicious, especially in the absence of an initial first contact with the research team. As well, while recruitment at community celebrations was effective for several communities, it was not so for the Chinese and West Balkan communities. For the former, recruitment in traditional medicine centres was more effective, and for the latter, an approach based on personal referrals worked best [Additional file 5]. It would be important to conduct studies upstream from the recruitment to identify what recruitment methods are appropriate for certain hard-to-reach communities (such as the West Balkans, Chinese, or South Asians). *“More studies should have been done on this population, to build a specific strategy and not waste our efforts”* [woman, Albania, II]. However, interviewers raised the importance of adapting recruitment strategies to individuals, regardless of their cultural affiliation. In fact, some people preferred to be interviewed by someone from outside their cultural or social community, whereas others preferred to be interviewed by members of their own community: *“It depends; they feel much more comfortable with someone from their community of origin, they can cover lots of topics. However, there’s a category of people who wouldn’t answer a questionnaire with someone from their community”* [woman, Algeria, II].

- Identifying and adapting recruitment strategies: a continuous and reflective process

In a context where a very heterogeneous population is targeted and where potential recruitment areas are diverse, regular reflective meetings and information sharing among all research team members (coordinator, assistants, trainees, researchers) are crucial to be able to continuously adapt and improve recruitment strategies to suit the contexts encountered. Such meetings were not always possible, because of budget constraints or team members’ unavailability. Several statements from the clusters “Leaders close to the field” [4.21; 3.91] and “A close-knit and engaged team of interviewers” [4.22; 3.80] [Additional file 1] highlight the importance of holding reflexive meetings with all team members, not only to provide feedback on complex cases encountered by the team, but also to allow interviewers to discuss the effectiveness of the strategies in the various contexts.

## Discussion

We have analyzed the specific challenges and strategies involved in recruiting and collecting data on migrants with precarious status. The strategies were defined upstream from the recruitment, in response to the obstacles identified in the literature on recruiting hard-to-reach populations, and some strategies also emerged during the recruitment process. The obstacles were mainly linguistic and cultural barriers [11, 13], people’s suspicions regarding research [55], and the lack of any perceived benefit from participating. After these strategies were implemented, new challenges emerged, uncovered by the present study: the difficulty of identifying the target population who, being very heterogeneous, are not easily distinguishable from other members of their community; the challenges of creating trusting relationships with community members (community organizations and places of worship); ethical challenges regarding appropriate compensation for participation; and cultural challenges related to matching the ethnocultural identities of the interviewer and the interviewee.

### Recruitment

First, there are ethical issues with regard to appropriate compensation for precarious status migrants’ participation in research. In our study, monetary compensation was not without controversy, whereas providing a guide to community resources led to effective and non-instrumental participation in research. While the amount paid out was intended to compensate participants for the time and effort invested in the survey [56], some communities in the present study perceived that compensation negatively. One study stated, in fact, that monetary compensation raised more suspicions among certain minority ethnic groups [57]. However, the majority of studies on ethnic minorities and migrants provide monetary compensation or non-financial alternatives (e.g. restaurant vouchers, subway tickets) [20], without putting participants in contact with resources suited to their needs. The development of a community resource guide tailored to the needs of participants [58] and the cultural appropriateness of incentives [20] should be major components of any research project targeting migrants. In addition, the study becomes an intervention in itself if interviewers redirect and accompany participants to resources, which was done in particularly vulnerable cases. The research team needs to consider the ethical dilemmas in which interviewers can be placed. This is particularly the case if they receive certain requests from participants, when there are not always resources available, because this population is often excluded from the services offered by aid organizations.

Second, in our study one challenge was to build stable links of trust with community organizations**.** Partnering with community organizations to sample target populations has been identified in the literature as useful for accessing the target population [13, 30, 59], and for overcoming the suspicions of hard-to-reach persons towards research [55]. However, in the present study, it was not always easy to build relationships of trust with all the community organizations and to fit exchange relationships into the time available. Indeed, some authors stress that participatory action research requires not only an investment of time and resources to establish relationships of trust with migrants and community organizations [30, 60], but also diplomacy and power-sharing [61]. In fact, it is difficult to have reciprocal exchange relationships when many organizations related to the different groups targeted by the study must be approached. While, in the present study, the community partnership approach could not therefore be sufficiently exhaustive to allow recruitment in all the targeted communities, it has proven effective in certain studies recruiting persons from three specific immigrant and refugee groups [62]. As such, this strategy may be better suited to projects targeting a particular geographic area or ethnocultural group. To be effective in a context of diversity, it is recommended that this approach be linked with recruitment in public spaces, as well as with a communication strategy. Some articles, in fact, confirm that using a diversity of approaches to contact vulnerable populations [63] and migrants [19] is essential for successful recruitment.

We cannot describe the effectiveness of the recruitment strategies without taking into account the infrastructural context described by Pawson [47] representing the social and cultural contexts. The various recruitment methods were effective when they were culturally adapted. This confirms the results of other studies, which have stressed the importance of translating the study materials into the languages of the targeted communities and of having the strategies implemented by a team that has the linguistic competency to connect with ethnic minorities [34, 64]. Moreover, some authors [65, 66] suggest that, to be accepted and thus effective, recruitment strategies need to reflect the behaviours, habits, and expectations of a group’s members. Even though a shared ethnocultural identity between interviewers and interviewees can sometimes reinforce trust [23, 67, 68], this strategy should not be systematic. In our study, matching the so-called ethnocultural identities of respondents and interviewers was not always appropriate. One study has shown that the perceptions, beliefs, and values of migrants who migrated at different times or who come from different social classes or educational backgrounds can vary considerably from one person to another. Thus, some participants preferred to share information with strangers who had no connection with the ethnocultural community [61]. Cultural homogeneity is an illusion that public health actors sometimes harbour [69]. In the present study, shared common experiences between the interviewers and participants were more relevant than cultural issues in fostering relationships of trust. This may have been particularly because sharing a similar life experience made it possible to overcome differences in social class, income, or education [30]. However, while some articles highlight the importance of having staff who are sensitive to the cultural and social dimensions of the people encountered [20], or are in contact with community organizations [62]*,* few articles mention the importance of recruiting interviewers with migratory pathways similar to those of the target population. It should be noted that this raises ethical challenges and power issues.

### Data collection and quality

The results of our study showed that the presence of an interviewer during the recruitment is crucial, not only to establish relationships of trust, in order to obtain a proper response rate to the questionnaire, but also to ensure good data quality. Training interviewers on the questionnaire is crucial to ensure their restating does not alter the meaning of the questions and thus to ensure the quality of data collection. This has been confirmed in certain studies conducted with ethnic minorities. In fact, the interviewer’s presence helped respondents to better understand the questions [34] and resulted in a higher response rate than is usually obtained in self-administered questionnaires in the case of one study conducted in areas of extreme precariousness [70]. Prior to data collection, collaboration between researchers and bilingual interviewers helps ensure accurate translation of the questionnaire, which is essential for its linguistic and cultural validity [71]*.*

### Additional resources for the recruitment of precarious status migrants

Implementing these recruitment strategies requires a significant budget. In our study, the time and resources needed for recruitment were underestimated, and it was calculated that an additional amount of 102,060 CAD, or 76% of the budget allocated for recruitment activities, would have been more expedient [44]. In the literature, the need for significant budgetary resources has been identified as a challenge specific to the recruitment of ethnic minorities. Indeed, recruiting a multicultural team, translating documents into the languages of the target communities, and having interviewers present during questionnaire administration requires a greater budget than is needed to recruit other hard-to-reach populations [34, 72, 73]*.*

### Recommendations for recruitment

Based on our study, we are able to propose some recommendations regarding strategies to be used in this type of research (Table 3). We believe these recommendations could also apply to other populations that are under-represented in research projects, particularly ethnic minorities.

**Table 3:** Main recommendations

(1) Identify recruitment strategies	• Interview key informants in the setting (focus groups and qualitative interviews) • Compile geographic information on the relevant recruitment areas • Conduct field studies to identify specific recruitment strategies for hard-to-reach communities • Use a variety of recruitment methods (social marketing, venue-based approaches, snowball sampling)
(2) Recruit interviewers	• Select a team that is diversified in terms of culture, migratory pathways, and gender • Hire interviewers who are connected to the network of uninsured migrants through their involvement in organizations or mutual support groups • Select interviewers who are experienced in connecting with vulnerable populations • Select interviewers who are familiar with the factors associated with migratory status
(3) Manage the project	• Encourage interviewers’ empowerment (by involving and consulting them) • Encourage the sharing of information and know-how among experienced and less experienced interviewers through regular informal and formal meetings • Set up a project coordination that is accessible and responsive to interviewers to foster relationships of trust • Encourage researchers’ involvement in field outings • Encourage regular reflexive meetings among all members of the project team
(4) Build relationships of trust with community members	• Establish collaborations with community organizations / places of worship / other organizations *before* starting recruitment • Develop a strategy to identify the team in the field and ensure the project’s visibility in community media • Ensure ongoing involvement and personalized follow-up with community members, transparency, and sharing of research results • Use key informants and “gate-keepers” in each community to reach the target population
(5) Adapt strategies to the target communities and individuals	• Give preference to cultural pairing for recruitment and let participants choose the interviewer with whom they would prefer to complete the questionnaire • Administer the questionnaire in the presence of an interviewer who can restate and explain certain questions when they are not understood and verify the consistency of the participant’s responses • Involve interviewers in selecting recruitment strategies, developing the questionnaire and the project information materials, and translating recruitment materials
(6) Take ethical issues into account in the recruitment	• Put participants in contact with community resources suited to their needs • Do not use words such as ‘study’ or ‘survey’ in the information materials but emphasize the help that can be provided by the project • Do not emphasize monetary compensation • Reassure participants by explaining to them that their data will remain anonymous and confidential • Train interviewers in the ethical and intercultural aspects of recruitment, as well as in the objectives and potential benefits of the study

### Methodological limitations

Only 39% of the total number of interviewers (*n* = 41) participated in this study. We were unfortunately unable to interview the interviewers who worked in community organizations or those who, due to conflicts, left the team. It should be emphasized that the data from the reflexive workshop are dependent on the social context in which the interactions took place. Certain dynamics may have contributed to the production of a dominant discourse that, as such, may not have been representative of the individual views of all participants [74]. Also, for reasons of social desirability, the interviewers may not have raised all the problems associated with the project’s management. Nevertheless, the preliminary results of the present study were presented to and validated by the interviewers during the conceptual mapping workshop. Lastly, it is important to keep in mind that only the interviewers’ perspectives were taken into account in analyzing the strategies, and that we did not consult the participants in the study, in contrast to recent studies in Europe [13] and in the United States of America [14].

## Conclusion

To implement specific and effective strategies, it is important to know the challenges researchers encounter when recruiting precarious status migrants. For recruitment to be successful, it appears that the focus should primarily be on individual, institutional, and infrastructural strategies. Interviewers’ intrinsic qualities (openness, understanding of the migrant situation, human qualities) are fundamental to creating trusting relationships. The interviewers’ experience and their understanding of the issue are therefore important factors to take into consideration in future research. Moreover, the development of a community resource guide tailored to the needs of participants should be major components of any research project targeting migrants. Finally, strategies that are culturally adapted and that respect ethical principles should be implemented as the result of a continuous reflexive process among all members of the research team, to optimize the expertise of the researchers and the interviewers and to continuously improve the recruitment process. Given the limited data available on the situation of migrants, the International Organization for Migration (IOM) is encouraging research to influence policy decisions [75, 76]. The World Health Organization (WHO) has also recommended the production of knowledge on the health situation of migrants and refugees, to promote the development of inclusive policies to support migrants [77]. From this standpoint, sharing the experiences and lessons learned by interviewers can offer a useful tool for future research projects, with a view to increasing the representation of migrants in research. It would be advisable for future studies to analyze the effects of recruitment strategies from the perspective of study participants and in these different contexts.

## Additional files

Additional file 1: Items scores. This table presents the names and scores of the different items from the concept mapping. (DOCX 27 kb)

Additional file 2: Clusters scores. This table presents names of the 8 clusters and their relevance and feasibility scores. These data come from the concept mapping. (DOCX 12 kb)

Additional file 3: The 10 least relevant statements and their feasibility scores. This table presents the 10 least relevant items and their feasibility scores. These data come from the concept mapping. (DOCX 13 kb)

Additional file 4: The 10 most relevant items and their feasibility scores. This table presents the 10 most relevant items and their feasibility scores. These data come from the concept mapping. (DOCX 13 kb)

Additional file 5: Effective strategies to reach communities. This table presents the most effective strategies for the different communities. (DOCX 17 kb)

## Abbreviations

CRCHUM: University of Montreal Hospital Research Centre
II: Individual interviews
IOM: International Organization for Migration
RW: Reflexive workshop
WHO: World Health Organization
