# Supplementary material for: Barriers and recruitment strategies for precarious status migrants in Montreal, Canada
Source: BMC Med Res Methodol. 2019 Feb 26;19:41. doi: 10.1186/s12874-019-0683-2 (PMC6390306; doi:10.1186/s12874-019-0683-2)
Supplement: Supplementary file 1 — Items scores. This table presents the names and scores of the different items from the concept mapping. (DOCX 27 kb) [file 12874_2019_683_MOESM1_ESM.docx]

| **Item** | **Relevance score** | **Feasibility**  **score** | **Cluster** |
| --- | --- | --- | --- |
| 1 Be empathetic and listen to people | 5 | 4.8 | Expert and committed interviewers |
| 74Recruit investigators with a view to working as a team | 4,9 | 3,6 |  |
| 20-Be able to adapt your speech according to the people | 4,9 | 4,4 |  |
| 21- Be able to be flexible in the field at the time of recruitment | 4,8 | 4,2 |  |
| 72- Have interviewers be sensitive to the interpersonal/psychosocial aspects of interviewers | 4,8 | 3,8 |  |
| 3 Choose engaged and committed interviewers | 4,7 | 3,6 |  |
| 23- To have skills to adjust to people and contexts | 4,67 | 3,44 |  |
| 4-Recruit interviewers who believe/are dedicated to the project’s objective | 4,6 | 3,3 |  |
| 70-Highlight the social and interpersonal skills of assistants | 4,5 | 3,8 |  |
| 73- To have interviewers with emotional intelligence | 4,5 | 3,2 |  |
| 7- Hire team members who speak multiple languages and from multiple communities | 4,5 | 3,8 |  |
| 67- Have assistants agree to work weekends and evenings | 4,4 | 3,6 |  |
| 71-Recruit assistants with psychosocial skills | 4,3 | 3,8 |  |
| 2 Choose the right team | 4,3 | 3,1 |  |
| 18- Recruit assistants with skills to involve migrants | 4,3 | 3,2 |  |
| 80- Recruit research assistants who are familiar with the issue and research area | 4,2 | 3,2 |  |
| 66- Choose research assistants who are available | 4,2 | 3,1 |  |
| 9- To have a good knowledge of the policies for which advocacy is sought | 4,1 | 3,9 |  |
| 62- Have investigators who know community resources | 4,1 | 3,4 |  |
| 61- Recruit investigators with experience with vulnerable communities | 4 | 3,4 |  |
| 41… that all investigators must have the same research discourse | 3,6 | 2,8 |  |
| 68- Identify/name key research principles for recruiting assistants | 3,4 | 3,6 |  |
| 8-Establish a personal connection with research participants | 2,6 | 2,3 |  |
| 60- Review interviewers' backgrounds before hiring them | 2,5 | 1,8 |  |
| 13- To teach interviewers to distance themselves from their prejudices towards participants | 4,9 | 3,5 | Unified and committed team |
| 76- That interviewers know how the results will be used to be able to better explain to participants | 4,5 | 4,4 |  |
| 57- Organize regular reflexive meetings between assistants | 4,4 | 4,3 |  |
| 52- Train and educate investigators on research ethics | 4,4 | 4,4 |  |
| 78- Have a strategy for sharing preliminary results with interviewers | 4,3 | 4,33 |  |
| 25- Involve future interviewers in developing the questionnaire | 4,2 | 3 |  |
| 16- Hold weekly meetings in the presence of research assistants | 4,1 | 3,1 |  |
| 14- Regularly monitor the interviewers regarding the completion of the questionnaires | 4 | 3,1 |  |
| 58- Organize regular psychological support sessions for assistants | 3,9 | 3,7 |  |
| 94- Organize social activities to strengthen links between research investigators | 3,9 | 4,1 |  |
| 69- Prioritize key research principles | 3,8 | 3,9 |  |
| 11- Collaborate with all community organizations in relation to participants | 4,8 | 3,4 | Recruitment tailored to settings and communities |
| 79- Develop partnership relationships with organizations in the health field to help participants | 4,78 | 3,11 |  |
| 59- Partner with multiple organizations | 4,7 | 3,4 |  |
| 19- Develop community-specific strategies (especially for hard-to-reach communities) | 4,7 | 3,8 |  |
| 10- To know settings where to find migrants | 4,5 | 3,6 |  |
| 33- Involve key participants as facilitators | 4,3 | 3,7 |  |
| 12- Invest time and resources with religious institutions/cults/community organizations | 4,3 | 3,4 |  |
| 32- Use gatekeepers in each community to access the community | 4,2 | 3,2 |  |
| 85- Have strategies that encourage participants to go to fixed locations | 4,2 | 3,4 |  |
| 31- Identify key participants (important in the community) | 4,1 | 3,2 |  |
| 75- Promote word of mouth to recruit participants | 4,1 | 3,5 |  |
| 84- Have several fixed locations to administer the questionnaire | 4,1 | 3,3 |  |
| 87-Have a formal approach for certain places (e.g. places of worship) | 4,1 | 3,7 |  |
| 6- Hiring community leaders | 4,1 | 3,5 |  |
| 5- Find placement networks/agencies that work with people | 3,6 | 3,5 |  |
| 45- Establish contacts with official representative authorities (consulates/embassies) | 3,2 | 3 |  |
| 53- Find an effective strategy to reach consulates | 3,1 | 3,5 |  |
| 82- Have a plan B for each recruitment site | 4,6 | 4,1 | Adaptability and motivation in the field |
| 89- Be flexible in terms of field presence | 4,6 | 4 |  |
| 48- Have a motivation strategy in the field | 4,4 | 3,8 |  |
| 15- Foster creativity in interviewing and recruitment | 4,3 | 3,6 |  |
| 90- Be consistent in field presence | 4,3 | 3,5 |  |
| 83- Fostering cultural pairs for recruitment | 3,78 | 4,22 |  |
| 39- Better present the fact sheet from a marketing perspective | 5 | 4,7 | Social marketing of the study |
| 40 Better define the resource support provided by the research in the fact sheet | 4,89 | 4,33 |  |
| 56 Put messages in several languages accessible on the Internet | 4,8 | 4,2 |  |
| 91- To promote the project | 4,8 | 4,3 |  |
| 77- That the project explains how the results will be used to improve people's lives | 4,8 | 4,2 |  |
| 55- Have the same search message in multiple languages | 4,6 | 4,4 |  |
| 43- Have a marketing strategy | 4,5 | 4,1 |  |
| 49- Have a communication strategy in the field | 4,5 | 4 |  |
| 47- Organize an all-out campaign before interwiewers are in the field | 4,3 | 4 |  |
| 17- Have the support of a marketing expert to facilitate communication | 4,1 | 4 |  |
| 42- Have an official speech that can be adapted to different communities | 4,1 | 3,8 |  |
| 88- Use websites (Kijiji, job site) used by participants for recruitment | 4 | 4 |  |
| 22- Involve communities more through community radio | 3,8 | 3,4 |  |
| 92- Use social networks instead of the website | 3,7 | 3,7 |  |
| 54- Make vox pop (record videos of research assistants) with all research assistants | 3,7 | 3,7 |  |
| 44- Create a central research concept/slogan but adapted to each community | 3,6 | 3,9 |  |
| 46- Have a telephone to be reachable at any times | 4,8 | 4,5 | Well-being and protection of the interviewers |
| 81- Provide identification elements related to the project (caps, cards, badges, etc.) at the beginning of the research | 4,1 | 4,1 |  |
| 93- Provide water to interviewers in the field | 3,8 | 4,7 |  |
| 35- Organizing festive activities | 3,3 | 3 |  |
| 24- Restate the questions to make them accessible to the participant/investigator | 4,7 | 4,3 | To be concerned with participants |
| 34- Give practical tools to participants | 4,6 | 4,1 |  |
| 63- Have professional equipment/logistics to attract participants | 4,6 | 4,4 |  |
| 36- Give small resource guides | 4,2 | 3,9 |  |
| 50- Use a reference guide with community organizations | 4,2 | 3,7 |  |
| 38- Have resource guides for education | 3,5 | 3,8 |  |
| 37- Organize a resource guide by needs | 3,4 | 3,1 |  |
| 86- Use a variety of means to complete the questionnaire (telephone, internet) | 3,2 | 3,6 |  |
| 27- Involve researchers at the first outing to be aware of the reality in the field | 4,7 | 4,3 | Managers of the program working closely with the field |
| 26- Involving researchers in the field from the beginning of the recruitment | 4,7 | 3,8 |  |
| 65-Have a culturally sensitive and multilingual administration | 4,5 | 3,5 |  |
| 29-Have a culturally sensitive and responsive administration | 4,3 | 4,1 |  |
| 51- Clearly define the role of the field coordinator | 4,1 | 4,7 |  |
| 64- Have a coordination that already has close ties with community organizations | 4 | 3,6 |  |
| 28-To have a professional administration expert in research | 3,8 | 3,5 |  |
| 30- To hire a Resource Administration Advisor | 3,6 | 3,8 |  |
