# Supplementary material for: Barriers and recruitment strategies for precarious status migrants in Montreal, Canada
Source: BMC Med Res Methodol. 2019 Feb 26;19:41. doi: 10.1186/s12874-019-0683-2 (PMC6390306; doi:10.1186/s12874-019-0683-2)
Supplement: Supplementary file 2 — Clusters scores. This table presents names of the 8 clusters and their relevance and feasibility scores. These data come from the concept mapping. (DOCX 12 kb) [file 12874_2019_683_MOESM2_ESM.docx]

| Cluster | Relevance score | Feasibility score |
| --- | --- | --- |
| Adaptability and motivation in the field | 4,33 | 3,87 |
| Social marketing of the study | 4,3 | 4,05 |
| Expert and committed interviewers | 4,24 | 3,46 |
| Unified and committed team | 4,22 | 3,80 |
| Managers of the program working closely with the field | 4,21 | 3,91 |
| Recruitment tailored to settings and communities | 4,17 | 3,42 |
| To be concerned with participants | 4,05 | 3,86 |
| Well-being and protection of the interviewers | 4,00 | 4,38 |
