# Supplementary material for: Barriers and recruitment strategies for precarious status migrants in Montreal, Canada
Source: BMC Med Res Methodol. 2019 Feb 26;19:41. doi: 10.1186/s12874-019-0683-2 (PMC6390306; doi:10.1186/s12874-019-0683-2)
Supplement: Supplementary file 3 — The 10 least relevant statements and their feasibility scores. This table presents the 10 least relevant items and their feasibility scores. These data come from the concept mapping. (DOCX 13 kb) [file 12874_2019_683_MOESM3_ESM.docx]

| **Item** | **Cluster** | **Relevance score** | **Feasibility score** |
| --- | --- | --- | --- |
| To hire a Resource Administration Advisor | Managers of the program working closely with the field | 3.6 | 3.8 |
| Have resource guides for education | To be concerned with participants | 3.5 | 3.8 |
| Identify/name key research principles for recruiting assistants | Expert and committed interviewers | 3.4 | 3.6 |
| Organize a resource guide by needs | To be concerned with participants | 3.4 | 3.4 |
| Organizing festive activities | Well-being and protection of the interviewers | 3.3 | 3 |
| Establish contacts with official representative authorities (consulates/embassies | Recruitment tailored to settings and communities | 3.2 | 3 |
| Use a variety of means to complete the questionnaire (telephone, internet) | To be concerned with participants | 3.2 | 3.6 |
| Find an effective strategy to reach consulates | Recruitment tailored to settings and communities | 3.1 | 3.5 |
| Establish a personal connection with research participants | Expert and committed interviewers | 2.6 | 2.3 |
| Review investigators' backgrounds before hiring them | Expert and committed interviewers | 2.5 | 1.8 |
