# Supplementary material for: Barriers and recruitment strategies for precarious status migrants in Montreal, Canada
Source: BMC Med Res Methodol. 2019 Feb 26;19:41. doi: 10.1186/s12874-019-0683-2 (PMC6390306; doi:10.1186/s12874-019-0683-2)
Supplement: Supplementary file 4 — The 10 most relevant items and their feasibility scores. This table presents the 10 most relevant items and their feasibility scores. These data come from the concept mapping. (DOCX 13 kb) [file 12874_2019_683_MOESM4_ESM.docx]

| **Item** | **Cluster** | **Relevance score** | **Feasibility score** |
| --- | --- | --- | --- |
| Be empathetic and listen to people | Expert and committed interviewers | 5 | 4.8 |
| Better present the fact sheet from a marketing perspective | Social marketing of the study | 5 | 4.7 |
| Recruit interviewers with a view to working as a team | Expert and committed interviewers | 4.9 | 3,6 |
| Be able to adapt your speech according to the people | To be concerned with participants | 4.9 | 4.4 |
| To teach interviewers to distance themselves from their prejudices towards participants | Unified and committed team | 4.9 | 3.5 |
| Better define the resource support provided by the research in the fact sheet | Social marketing of the study | 4.89 | 4.33 |
| Have interviewers be sensitive to the interpersonal/psychosocial aspects of interviewers | Expert and committed interviewers | 4.8 | 3.7 |
| Be able to be flexible in the field at the time of recruitment | Expert and committed interviewers | 4.8 | 4.2 |
| Collaborate with all community organizations in relation to participants | Recruitment tailored to settings and communities | 4.8 | 3.4 |
| Put messages in several languages accessible on the Internet | Social marketing of the study | 4.8 | 4.2 |
