# Supplementary material for: Barriers and recruitment strategies for precarious status migrants in Montreal, Canada
Source: BMC Med Res Methodol. 2019 Feb 26;19:41. doi: 10.1186/s12874-019-0683-2 (PMC6390306; doi:10.1186/s12874-019-0683-2)
Supplement: Supplementary file 5 — Effective strategies to reach communities. This table presents the most effective strategies for the different communities. (DOCX 17 kb) [file 12874_2019_683_MOESM5_ESM.docx]

| **Communities** | **Strategies used** |
| --- | --- |
| Maghrebin | Held community coffee meets.  Chose an interviewer who was not too close to this community.  Approached people through places of worship. |
| West Balkan | Sought out personal references.  Identified and established relationships of trust with community informants. |
| Chinese | Approached people through the traditional Chinese medicine centre.  Recruited in Chinatown, international student associations, and the Chinese Family Community Centre.  Approached people through the food bank and at social events. |
| Haitian | Sought out personal references.  Used snowball sampling.  Approached people through places of worship. |
| Middle Eastern | Presented the humanitarian aspect of the project by explaining the community resources of the project and its potential socio-economic benefits over the long term. |
| African (sub-Saharan) | Interviewers presented themselves as staff of African origin who fought for people without medical coverage.  Worked hard to raise people’s awareness on issues that sometimes concerned those around them. |
| Latin-American | Approached people through community events.  Used social media such as Facebook and Kijiji. |
| South Asian | Approached people through community events.  Identified and established relationships of trust with community informants.  Approached people at religious events and places of worship. |
| Russian | Approached people in public places  Approached people via the fair solutions clinic |
| American (United States) | Approached people in public places, urban areas |
| European | Approached people in public places  Worked with community organizations  For the French community, approached people during the presidential elections  Presented the project to community organizations, sports clubs, and the French consulate. |
| Anglo-Caribbean | Difficulties creating links with the community due to lack of community members on the research team to establish trusting relationships. |
| All communities combined | Spoke about community services to attract the attention of the people encountered.  Took time to talk to people and targeted their needs (letting people express themselves and react). |

Source: Reflection tools of the reflexive workshop, and the report on recruitment strategies
